# Supplementary material for: An analysis of behavioral characteristics and enrollment year variability in 47,444 dogs entering the Dog Aging Project from 2020 to 2023
Source: PLoS One. 2025 Sep 10;20(9):e0330257. doi: 10.1371/journal.pone.0330257 (PMC12422430; doi:10.1371/journal.pone.0330257)
Supplement: S1 File — S1 Table. Results of regression analyses examining the relationships between variables of interest and mean behavior scores for dogs in four composite behavior domains: PC1 – Fear; PC2 – Attention; PC3 – Aggression; PC4 – Trainability. Dog Aging Project, 2020–2023. S2 Table. Results of initial regression models including interaction terms examining the relationships between variables of interest and mean behavior scores for dogs in four composite behavior domains: PC1 – Fear; PC2 – Attention; PC3 – Aggression; PC4 – Trainability. Dog Aging Project, 2020–2023. (PDF) [file pone.0330257.s001.pdf]

**S1 Table.** Results of regression analyses examining the relationships between variables of interest and mean behavior scores for dogs in four composite behavior domains: PC1 - Fear; PC2 - Attention; PC3 - Aggression; PC4 - Trainability. Dog Aging Project, 2020–2023.

## PRINCIPAL COMPONENT

### 1. FEAR

| Variable | | N | Estimate | Standard Error | P-value |
|---|---|---|---|---|---|
| **Year** | 2020 *(reference)* | 27437 | | | |
| | 2021 | 5653 | 0.004 | 0.014 | 0.800 |
| | 2022 | 10379 | -0.008 | 0.011 | 0.460 |
| | 2023 | 3975 | -0.009 | 0.016 | 0.567 |
| **Breed** | Mixed *(reference)* | 23587 | | | |
| | Single | 23875 | -0.303 | 0.009 | <0.001* |
| **Life Stage at Health and Life Experience Survey (HLES)** | Puppy *(reference)* | 2525 | | | |
| | Young Adult | 10091 | 0.158 | 0.023 | <0.001* |
| | Mature Adult | 26271 | 0.266 | 0.023 | <0.001* |
| | Senior | 8556 | 0.140 | 0.025 | <0.001* |
| **Sex** | Male *(reference)* | 23890 | | | |
| | Female | 23554 | 0.106 | 0.009 | <0.001* |
| **Spay/Neuter Status** | Intact *(reference)* | 5556 | | | |
| | Spayed/Neutered | 41888 | 0.274 | 0.016 | <0.001* |
| **Size (weight class)** | < 20lbs (~9kg) *(reference)* | 9464 | | | |
| | 21-40lbs (~10-18kg) | 9122 | -0.195 | 0.014 | <0.001* |
| | 41-60lbs (~19-27kg) | 12062 | -0.260 | 0.013 | <0.001* |
| | 61-80lbs (~28-36kg) | 10593 | -0.359 | 0.014 | <0.001* |
| | 81-100lbs (~37-45kg) | 4097 | -0.324 | 0.018 | <0.001* |
| | >100lbs (~45kg) | 2106 | -0.248 | 0.023 | <0.001* |
| **Health Insurance Status** | No *(reference)* | 37317 | | | |
| | Yes | 10127 | -0.020 | 0.011 | 0.068 |

| | | | | | |
|---|---|---|---|---|---|
| **U.S. Region of Residence** | Midwest *(reference)* | 9219 | | | |
| | Northeast | 7690 | -0.011 | 0.015 | 0.446 |
| | South | 14114 | -0.004 | 0.013 | 0.760 |
| | West | 16421 | 0.009 | 0.013 | 0.479 |
| **Primary Activities** | Companion animal or pet *(reference)* | 45036 | | | |
| | Assistance or therapy dog | 335 | -0.143 | 0.053 | 0.007* |
| | Obedience | 354 | 0.013 | 0.053 | 0.804 |
| | Service Dog | 462 | -0.483 | 0.045 | <0.001* |
| | Other | 1257 | -0.069 | 0.028 | 0.013* |

## 2. ATTENTION

| Variable | | N | Estimate | Standard Error | P-value |
|---|---|---|---|---|---|
| **Year** | 2020 (*reference*) | 27437 | | | |
| | 2021 | 5653 | 0.023 | 0.013 | 0.091 |
| | 2022 | 10379 | 0.023 | 0.011 | 0.031* |
| | 2023 | 3975 | 0.018 | 0.016 | 0.239 |
| **Breed** | Mixed *(reference)* | 23587 | | | |
| | Single | 23875 | -0.047 | 0.009 | <0.001* |
| **Life Stage at Health and Life Experience Survey (HLES)** | Puppy *(reference)* | 2525 | | | |
| | Young Adult | 10091 | -0.254 | 0.022 | <0.001* |
| | Mature Adult | 26271 | -0.855 | 0.022 | <0.001* |
| | Senior | 8556 | -0.124 | 0.023 | <0.001* |
| **Sex** | Male *(reference)* | 23890 | | | |
| | Female | 23554 | -0.135 | 0.009 | <0.001* |
| **Spay/Neuter Status** | Intact *(reference)* | 5556 | | | |
| | Spayed/Neutered | 41888 | -0.074 | 0.015 | <0.001* |
| **Size (weight class)** | < 20lbs (~9kg) *(reference)* | 9464 | | | |
| | 21-40lbs (~10-18kg) | 9122 | -0.112 | 0.013 | <0.001* |

| | | | | | |
|---|---|---|---|---|---|
| | 41-60lbs (~19-27kg) | 12062 | -0.177 | 0.013 | <0.001* |
| | 61-80lbs (~28-36kg) | 10593 | -0.248 | 0.013 | <0.001* |
| | 81-100lbs (~37-45kg) | 4097 | -0.286 | 0.017 | <0.001* |
| | >100lbs (~45kg) | 2106 | -0.545 | 0.022 | <0.001* |
| **Health Insurance Status** | No *(reference)* | 37317 | | | |
| | Yes | 10127 | 0.017 | 0.011 | 0.098 |
| **U.S. Region of Residence** | Midwest *(reference)* | 9219 | | | |
| | Northeast | 7690 | -0.038 | 0.014 | 0.007* |
| | South | 14114 | -0.024 | 0.012 | 0.048* |
| | West | 16421 | -0.071 | 0.012 | <0.001* |
| **Primary Activities** | Companion animal or pet *(reference)* | 45036 | | | |
| | Assistance or therapy dog | 335 | -0.095 | 0.050 | 0.092 |
| | Obedience | 354 | -0.083 | 0.049 | 0.091 |
| | Service Dog | 462 | -0.363 | 0.043 | <0.001* |
| | Other | 1257 | -0.143 | 0.026 | <0.001* |

## 3. AGGRESSION

| Variable | | N | Estimate | Standard Error | P-value |
|---|---|---|---|---|---|
| **Year** | 2020 *(reference)* | 27437 | | | |
| | 2021 | 5653 | 0.006 | 0.014 | 0.680 |
| | 2022 | 10379 | -0.005 | 0.012 | 0.685 |
| | 2023 | 3975 | -0.090 | 0.017 | <0.001* |
| **Breed** | Mixed *(reference)* | 23587 | | | |
| | Single | 23875 | -0.151 | 0.009 | <0.001* |
| **Life Stage at Health and Life Experience Survey (HLES)** | Puppy *(reference)* | 2525 | | | |
| | Young Adult | 10091 | 0.243 | 0.023 | <0.001* |
| | Mature Adult | 26272 | 0.499 | 0.023 | <0.001* |
| | Senior | 8556 | 0.497 | 0.025 | <0.001* |
| **Sex** | Male *(reference)* | 23890 | | | |

| | | | | | |
|---|---|---|---|---|---|
| | Female | 23554 | -0.051 | 0.009 | <0.001* |
| **Spay/Neuter Status** | Intact *(reference)* | 5556 | | | |
| | Spayed/Neutered | 41888 | 0.049 | 0.016 | 0.003* |
| **Size (weight class)** | < 20lbs (~9kg) *(reference)* | 9464 | | | |
| | 21-40lbs (~10-18kg) | 9122 | -0.099 | 0.014 | <0.001* |
| | 41-60lbs (~19-27kg) | 12062 | -0.228 | 0.014 | <0.001* |
| | 61-80lbs (~28-36kg) | 10593 | -0.283 | 0.014 | <0.001* |
| | 81-100lbs (~37-45kg) | 4097 | -0.233 | 0.018 | <0.001* |
| | >100lbs (~45kg) | 2106 | -0.188 | 0.024 | <0.001* |
| **Health Insurance Status** | No *(reference)* | 37317 | | | |
| | Yes | 10127 | -0.060 | 0.011 | 0.068 |
| **U.S. Region of Residence** | Midwest *(reference)* | 9219 | | | |
| | Northeast | 7690 | -0.031 | | 0.043* |
| | South | 14114 | -0.011 | | 0.420 |
| | West | 16421 | -0.050 | | <0.001* |
| **Primary Activities** | Companion animal or pet *(reference)* | 45036 | | | |
| | Assistance or therapy dog | 335 | -0.175 | 0.054 | 0.001* |
| | Obedience | 354 | -0.006 | 0.052 | 0.903 |
| | Service Dog | 462 | -0.314 | 0.046 | <0.001* |
| | Other | 1257 | 0.027 | 0.028 | 0.344 |

## 4. TRAINABILITY

| **Variable** | | **N** | **Estimate** | **Standard Error** | **P-value** |
|---|---|---|---|---|---|
| **Year** | 2020 *(reference)* | 27437 | | | |
| | 2021 | 5653 | -0.057 | 0.014 | <0.001* |
| | 2022 | 10379 | -0.054 | 0.011 | <0.001* |
| | 2023 | 3975 | -0.009 | 0.016 | <0.001* |
| **Breed** | Mixed *(reference)* | 23587 | | | |

| | | | | | |
|---|---|---|---|---|---|
| | Single | 23875 | -0.012 | 0.009 | 0.185 |
| **Life Stage at Health and Life Experience Survey (HLES)** | Puppy *(reference)* | 2525 | | | |
| | Young Adult | 10091 | 0.437 | 0.023 | <0.001* |
| | Mature Adult | 26272 | 0.423 | 0.022 | <0.001* |
| | Senior | 8556 | -0.068 | 0.024 | 0.005* |
| **Sex** | Male *(reference)* | 23890 | | | |
| | Female | 23554 | 0.106 | 0.009 | <0.001* |
| **Spay/Neuter Status** | Intact *(reference)* | 5556 | | | |
| | Spayed/Neutered | 41888 | -0.046 | 0.016 | 0.004* |
| **Size (weight class)** | < 20lbs (~9kg) *(reference)* | 9464 | | | |
| | 21-40lbs (~10-18kg) | 9122 | 0.294 | 0.014 | <0.001* |
| | 41-60lbs (~19-27kg) | 12062 | 0.478 | 0.013 | <0.001* |
| | 61-80lbs (~28-36kg) | 10593 | 0.510 | 0.014 | <0.001* |
| | 81-100lbs (~37-45kg) | 4097 | 0.553 | 0.018 | <0.001* |
| | >100lbs (~45kg) | 2106 | 0.378 | 0.023 | <0.001* |
| **Health Insurance Status** | No *(reference)* | | | | |
| | Yes | 10127 | 0.016 | 0.011 | 0.149 |
| **U.S. Region of Residence** | Midwest *(reference)* | 9219 | | | |
| | Northeast | 7690 | -0.052 | 0.015 | <0.001* |
| | South | 14114 | -0.049 | 0.013 | <0.001* |
| | West | 16421 | -0.006 | 0.012 | 0.630 |
| **Primary Activities** | Companion animal or pet *(reference)* | 45036 | | | |
| | Assistance or therapy dog | 335 | 0.003 | 0.052 | 0.958 |
| | Obedience | 354 | 0.196 | 0.051 | <0.001* |
| | Service Dog | 462 | -0.074 | 0.045 | 0.097 |
| | Other | 1257 | 0.100 | 0.027 | <0.001* |

**Page intentionally left blank.**

**S2 Table.** Results of initial regression models including interaction terms examining the relationships between variables of interest and mean behavior scores for dogs in four composite behavior domains: PC1 - Fear; PC2 - Attention; PC3 - Aggression; PC4 - Trainability. Dog Aging Project, 2020–2023.

**PRINCIPAL COMPONENT**

**1. FEAR**

| Variable | | N | Estimate | Standard Error | P-value |
|---|---|---|---|---|---|
| **Year** | 2020 *(reference)* | 27437 | | | |
| | 2021 | 5653 | -0.068 | 0.077 | 0.376 |
| | 2022 | 10379 | -0.096 | 0.064 | 0.120 |
| | 2023 | 3975 | -0.065 | 0.088 | 0.462 |
| **Breed** | Mixed *(reference)* | 23587 | | | |
| | Single | 23875 | -0.294 | 0.121 | <0.001* |
| | Single:2021 | | -0.006 | 0.030 | 0.851 |
| | Single:2022 | | -0.032 | 0.023 | 0.168 |
| | Single:2023 | | -0.020 | 0.034 | 0.568 |
| **Life Stage at Health and Life Experience Survey (HLES)** | Puppy *(reference)* | 2525 | | | |
| | Young Adult | 10091 | 0.069 | 0.044 | 0.115 |
| | Mature Adult | 26271 | 0.181 | 0.043 | <0.001* |
| | Senior | 8556 | 0.063 | 0.044 | 0.158 |
| | Young Adult:2021 | | 0.115 | 0.070 | 0.098 |
| | Young Adult:2022 | | 0.136 | 0.058 | 0.018* |
| | Young Adult:2023 | | 0.100 | 0.078 | 0.203 |
| | Mature Adult:2021 | | 0.110 | 0.067 | 0.100 |
| | Mature Adult:2022 | | 0.104 | 0.057 | 0.070 |
| | Mature Adult:2023 | | 0.147 | 0.077 | 0.057 |
| | Senior:2021 | | 0.061 | 0.073 | 0.407 |
| | Senior:2022 | | 0.132 | 0.063 | 0.037* |
| | Senior:2023 | | 0.068 | 0.085 | 0.423 |
| **Sex** | Male *(reference)* | 23890 | | | |
| | Female | 23554 | 0.098 | 0.012 | <0.001* |
| | Female:2021 | | 0.011 | 0.029 | 0.691 |
| | Female:2022 | | 0.008 | 0.023 | 0.734 |
| | Female:2023 | | 0.057 | 0.033 | 0.083 |

| | | | | | |
|---|---|---|---|---|---|
| **Spay/Neuter Status** | Intact *(reference)* | 5556 | | | |
| | Spayed/Neutered | 41888 | 0.284 | 0.024 | <0.001* |
| | Spayed/Neutered:2021 | | -0.008 | 0.048 | 0.873 |
| | Spayed/Neutered:2022 | | -0.016 | 0.038 | 0.670 |
| | Spayed/Neutered:2023 | | -0.063 | 0.056 | 0.261 |
| **Size (weight class)** | < 20lbs (~9kg) *(reference)* | 9464 | | | |
| | 21-40lbs (~10-18kg) | 9122 | -0.201 | 0.019 | <0.001* |
| | 41-60lbs (~19-27kg) | 12062 | -0.257 | 0.018 | <0.001* |
| | 61-80lbs (~28-36kg) | 10593 | -0.376 | 0.018 | <0.001* |
| | 81-100lbs (~37-45kg) | 4097 | -0.341 | 0.024 | <0.001* |
| | >100lbs (~45kg) | 2106 | -0.257 | 0.031 | <0.001* |
| | 21-40lbs:2021 | | -0.040 | 0.046 | 0.391 |
| | 21-40lbs:2022 | | 0.025 | 0.035 | 0.484 |
| | 21-40lbs:2023 | | 0.061 | 0.052 | 0.235 |
| | 41-60lbs:2021 | | 0.002 | 0.043 | 0.958 |
| | 41-60lbs:2022 | | -0.009 | 0.034 | 0.789 |
| | 41-60lbs:2023 | | -0.012 | 0.048 | 0.795 |
| | 61-80lbs:2021 | | 0.006 | 0.045 | 0.887 |
| | 61-80lbs:2022 | | 0.070 | 0.035 | 0.044* |
| | 61-80lbs:2023 | | 0.008 | 0.056 | 0.869 |
| | 81-100lbs:2021 | | 0.034 | 0.056 | 0.546 |
| | 81-100lbs:2022 | | 0.065 | 0.047 | 0.167 |
| | 81-100lbs:2023 | | -0.022 | 0.068 | 0.741 |
| | >100lbs:2021 | | -0.015 | 0.068 | 0.824 |
| | >100lbs:2022 | | -0.004 | 0.061 | 0.951 |
| | >100lbs:2023 | | 0.142 | 0.092 | 0.1221 |
| **Health Insurance Status** | No *(reference)* | 37317 | | | |
| | Yes | 10127 | -0.012 | 0.015 | 0.453 |
| | Yes:2021 | | -0.027 | 0.035 | 0.445 |
| | Yes:2022 | | -0.018 | 0.027 | 0.502 |
| | Yes:2023 | | -0.008 | 0.038 | 0.835 |
| **U.S. Region of Residence** | Midwest *(reference)* | 9219 | | | |
| | Northeast | 7690 | -0.008 | 0.020 | 0.697 |
| | South | 14114 | 0.019 | 0.017 | 0.265 |
| | West | 16421 | 0.009 | 0.016 | 0.582 |
| | Northeast:2021 | | -0.017 | 0.050 | 0.741 |

| | | | | | |
|---|---|---|---|---|---|
| | Northeast:2022 | | -0.011 | 0.037 | 0.762 |
| | Northeast:2023 | | -0.011 | 0.055 | 0.839 |
| | South:2021 | | -0.041 | 0.042 | 0.332 |
| | South:2022 | | -0.042 | 0.033 | 0.203 |
| | South:2023 | | -0.102 | 0.049 | 0.038* |
| | West:2021 | | 0.007 | 0.041 | 0.857 |
| | West:2022 | | 0.005 | 0.032 | 0.869 |
| | West:2023 | | -0.031 | 0.049 | 0.523 |
| **Primary Activities** | Companion animal or pet *(reference)* | 45036 | | | |
| | Assistance or therapy dog | 335 | -0.172 | 0.071 | 0.016* |
| | Obedience | 354 | -0.004 | 0.072 | 0.651 |
| | Service Dog | 462 | -0.508 | 0.065 | <0.001* |
| | Other | 1257 | -0.023 | 0.039 | 0.555 |
| | Assistance or therapy dog:2021 | | 0.075 | 0.166 | 0.652 |
| | Assistance or therapy dog:2022 | | 0.067 | 0.133 | 0.614 |
| | Assistance or therapy dog:2023 | | 0.054 | 0.181 | 0.764 |
| | Obedience:2021 | | 0.019 | 0.163 | 0.907 |
| | Obedience:2022 | | -0.023 | 0.125 | 0.854 |
| | Obedience:2023 | | 0.151 | 0.169 | 0.372 |
| | Service Dog:2021 | | 0.066 | 0.137 | 0.631 |
| | Service Dog:2022 | | -0.007 | 0.108 | 0.948 |
| | Service Dog:2023 | | 0.165 | 0.156 | 0.291 |
| | Other:2021 | | -0.112 | 0.083 | 0.178 |
| | Other:2022 | | -0.125 | 0.067 | 0.061 |
| | Other:2023 | | 0.042 | 0.104 | 0.683 |

## 2. ATTENTION

| Variable | | N | Estimate | Standard Error | P-value |
|---|---|---|---|---|---|
| **Year** | 2020 *(reference)* | 27437 | | | |
| | 2021 | 5653 | 0.039 | 0.073 | 0.599 |
| | 2022 | 10379 | -0.056 | 0.061 | 0.351 |
| | 2023 | 3975 | -0.057 | 0.083 | 0.493 |
| **Breed** | Mixed *(reference)* | 23587 | | | |
| | Single | 23875 | -0.029 | 0.011 | 0.013* |

| | | | | | |
|---|---|---|---|---|---|
| | Single:2021 | | -0.042 | 0.028 | 0.145 |
| | Single:2022 | | -0.045 | 0.022 | 0.042* |
| | Single:2023 | | -0.025 | 0.066 | 0.211 |
| **Life Stage at Health and Life Experience Survey (HLES)** | Puppy *(reference)* | 2525 | | | |
| | Young Adult | 10091 | -0.261 | 0.041 | <0.001* |
| | Mature Adult | 26271 | -0.868 | 0.041 | <0.001* |
| | Senior | 8556 | -1.244 | 0.042 | <0.001* |
| | Young Adult:2021 | | -0.025 | 0.066 | 0.705 |
| | Young Adult:2022 | | 0.005 | 0.055 | 0.933 |
| | Young Adult:2023 | | 0.018 | 0.075 | 0.805 |
| | Mature Adult:2021 | | -0.028 | 0.066 | 0.658 |
| | Mature Adult:2022 | | 0.025 | 0.054 | 0.645 |
| | Mature Adult:2023 | | 0.031 | 0.074 | 0.677 |
| | Senior:2021 | | -0.075 | 0.070 | 0.280 |
| | Senior:2022 | | 0.010 | 0.060 | 0.869 |
| | Senior:2023 | | 0.076 | 0.008 | 0.349 |
| **Sex** | Male *(reference)* | 23890 | | | |
| | Female | 23554 | -0.145 | 0.011 | <0.001* |
| | Female:2021 | | 0.019 | 0.027 | 0.494 |
| | Female:2022 | | 0.043 | 0.022 | 0.048* |
| | Female:2023 | | -0.002 | 0.032 | 0.948 |
| **Spay/Neuter Status** | Intact *(reference)* | 5556 | | | |
| | Spayed/Neutered | 41888 | -0.117 | 0.023 | <0.001* |
| | Spayed/Neutered:2021 | | 0.102 | 0.046 | 0.025* |
| | Spayed/Neutered:2022 | | 0.075 | 0.036 | 0.039* |
| | Spayed/Neutered:2023 | | 0.015 | 0.053 | 0.775 |
| **Size (weight class)** | < 20lbs (~9kg) *(reference)* | 9464 | | | |
| | 21-40lbs (~10-18kg) | 9122 | -0.115 | 0.018 | <0.001* |
| | 41-60lbs (~19-27kg) | 12062 | -0.165 | 0.017 | <0.001* |
| | 61-80lbs (~28-36kg) | 10593 | -0.251 | 0.017 | <0.001* |
| | 81-100lbs (~37-45kg) | 4097 | -0.291 | 0.023 | <0.001* |
| | >100lbs (~45kg) | 2106 | -0.495 | 0.030 | <0.001* |
| | 21-40lbs:2021 | | -0.065 | 0.044 | 0.141 |
| | 21-40lbs:2022 | | 0.025 | 0.034 | 0.458 |
| | 21-40lbs:2023 | | 0.049 | 0.049 | 0.319 |
| | 41-60lbs:2021 | | -0.083 | 0.041 | 0.044* |
| | 41-60lbs:2022 | | -0.008 | 0.032 | 0.795 |
| | 41-60lbs:2023 | | -0.008 | 0.046 | 0.863 |

| | | | | | |
|---|---|---|---|---|---|
| | 61-80lbs:2021 | | -0.018 | 0.042 | 0.663 |
| | 61-80lbs:2022 | | -0.001 | 0.033 | 0.978 |
| | 61-80lbs:2023 | | 0.063 | 0.048 | 0.189 |
| | 81-100lbs:2021 | | -0.045 | 0.053 | 0.400 |
| | 81-100lbs:2022 | | 0.054 | 0.045 | 0.223 |
| | 81-100lbs:2023 | | -0.028 | 0.064 | 0.660 |
| | >100lbs:2021 | | -0.208 | 0.065 | 0.001* |
| | >100lbs:2022 | | -0.074 | 0.058 | 0.205 |
| | >100lbs:2023 | | -0.087 | 0.087 | 0.318 |
| **Health Insurance Status** | No *(reference)* | 37317 | | | |
| | Yes | 10127 | 0.012 | 0.015 | 0.427 |
| | Yes:2021 | | -0.021 | 0.033 | 0.534 |
| | Yes:2022 | | 0.019 | 0.025 | 0.444 |
| | Yes:2023 | | 0.032 | 0.036 | 0.370 |
| **U.S. Region of Residence** | Midwest *(reference)* | 9219 | | | |
| | Northeast | 7690 | -0.038 | 0.019 | 0.043* |
| | South | 14114 | -0.016 | 0.016 | 0.302 |
| | West | 16421 | -0.078 | 0.015 | <0.001* |
| | Northeast:2021 | | 0.014 | 0.048 | 0.775 |
| | Northeast:2022 | | -0.023 | 0.036 | 0.523 |
| | Northeast:2023 | | 0.045 | 0.053 | 0.397 |
| | South:2021 | | -0.036 | 0.040 | 0.369 |
| | South:2022 | | -0.016 | 0.031 | 0.609 |
| | South:2023 | | 0.012 | 0.047 | 0.796 |
| | West:2021 | | 0.014 | 0.039 | 0.723 |
| | West:2022 | | 0.009 | 0.031 | 0.762 |
| | West:2023 | | 0.034 | 0.047 | 0.459 |
| **Primary Activities** | Companion animal or pet *(reference)* | 45036 | | | |
| | Assistance or therapy dog | 335 | -0.102 | 0.068 | 0.131 |
| | Obedience | 354 | -0.132 | 0.068 | 0.054 |
| | Service Dog | 462 | -0.392 | 0.062 | <0.001* |
| | Other | 1257 | -0.081 | 0.037 | 0.027* |
| | Assistance or therapy dog:2021 | | 0.122 | 0.157 | 0.439 |
| | Assistance or therapy dog:2022 | | -0.075 | 0.126 | 0.553 |
| | Assistance or therapy dog:2023 | | 0.225 | 0.172 | 0.191 |
| | Obedience:2021 | | 0.339 | 0.155 | 0.029* |
| | Obedience:2022 | | 0.080 | 0.119 | 0.505 |
| | Obedience:2023 | | -0.120 | 0.161 | 0.456 |

| | | | | | |
|---|---|---|---|---|---|
| | Service Dog:2021 | | 0.021 | 0.130 | 0.870 |
| | Service Dog:2022 | | 0.055 | 0.102 | 0.589 |
| | Service Dog:2023 | | 0.106 | 0.148 | 0.473 |
| | Other:2021 | | -0.078 | 0.079 | 0.326 |
| | Other:2022 | | -0.156 | 0.064 | 0.014* |
| | Other:2023 | | -0.075 | 0.099 | 0.449 |

## 3. AGGRESSION

| Variable | | N | Estimate | Standard Error | P-value |
|---|---|---|---|---|---|
| **Year** | 2020 (*reference*) | 27437 | | | |
| | 2021 | 5653 | -0.288 | 0.046 | <0.001* |
| | 2022 | 10379 | -0.220 | 0.078 | 0.001* |
| | 2023 | 3975 | -0.343 | 0.065 | <0.001* |
| **Breed** | Mixed *(reference)* | 23587 | | | |
| | Single | 23875 | -0.155 | 0.012 | <0.001* |
| | Single:2021 | | 0.043 | 0.030 | 0.155 |
| | Single:2022 | | 0.005 | 0.024 | 0.832 |
| | Single:2023 | | -0.023 | 0.035 | 0.506 |
| **Life Stage at Health and Life Experience Survey (HLES)** | Puppy *(reference)* | 2525 | | | |
| | Young Adult | 10091 | 0.146 | 0.044 | 0.001* |
| | Mature Adult | 26272 | 0.401 | 0.043 | <0.001* |
| | Senior | 8556 | 0.399 | 0.045 | <0.001* |
| | Young Adult:2021 | | 0.148 | 0.071 | 0.037* |
| | Young Adult:2022 | | 0.104 | 0.059 | 0.076 |
| | Young Adult:2023 | | 0.130 | 0.080 | 0.102 |
| | Mature Adult:2021 | | 0.105 | 0.068 | 0.123 |
| | Mature Adult:2022 | | 0.131 | 0.058 | 0.024* |
| | Mature Adult:2023 | | 0.114 | 0.079 | 0.145 |
| | Senior:2021 | | 0.175 | 0.074 | 0.019* |
| | Senior:2022 | | 0.011 | 0.064 | 0.072 |
| | Senior:2023 | | 0.065 | 0.086 | 0.455 |
| **Sex** | Male *(reference)* | 23890 | | | |
| | Female | 23554 | -0.053 | 0.012 | <0.001* |
| | Female:2021 | | 0.003 | 0.029 | 0.930 |
| | Female:2022 | | -0.002 | 0.023 | 0.945 |
| | Female:2023 | | 0.029 | 0.034 | 0.386 |

| | | | | | |
|---|---|---|---|---|---|
| **Spay/Neuter Status** | Intact *(reference)* | 5556 | | | |
| | Spayed/Neutered | 41888 | 0.0.42 | 0.024 | 0.086 |
| | Spayed/Neutered:2021 | | 0.046 | 0.049 | 0.348 |
| | Spayed/Neutered:2022 | | 0.013 | 0.039 | 0.736 |
| | Spayed/Neutered:2023 | | -0.019 | 0.057 | 0.738 |
| **Size (weight class)** | < 20lbs (~9kg) *(reference)* | 9464 | | | |
| | 21-40lbs (~10-18kg) | 9122 | -0.125 | 0.019 | <0.001* |
| | 41-60lbs (~19-27kg) | 12062 | -0.264 | 0.018 | <0.001* |
| | 61-80lbs (~28-36kg) | 10593 | -0.330 | 0.018 | <0.001* |
| | 81-100lbs (~37-45kg) | 4097 | -0.294 | 0.024 | <0.001* |
| | >100lbs (~45kg) | 2106 | -0.204 | 0.031 | <0.001* |
| | 21-40lbs:2021 | | 0.022 | 0.047 | 0.639 |
| | 21-40lbs:2022 | | 0.067 | 0.036 | 0.063 |
| | 21-40lbs:2023 | | 0.103 | 0.052 | 0.048* |
| | 41-60lbs:2021 | | 0.063 | 0.044 | 0.147 |
| | 41-60lbs:2022 | | 0.085 | 0.034 | 0.013* |
| | 41-60lbs:2023 | | 0.101 | 0.049 | 0.037* |
| | 61-80lbs:2021 | | 0.119 | 0.045 | 0.009* |
| | 61-80lbs:2022 | | 0.106 | 0.035 | 0.003* |
| | 61-80lbs:2023 | | 0.116 | 0.051 | 0.023* |
| | 81-100lbs:2021 | | 0.078 | 0.057 | 0.168 |
| | 81-100lbs:2022 | | 0.194 | 0.048 | <0.001* |
| | 81-100lbs:2023 | | 0.130 | 0.069 | 0.057 |
| | >100lbs:2021 | | -0.058 | 0.069 | 0.399 |
| | >100lbs:2022 | | 0.089 | 0.062 | 0.148 |
| | >100lbs:2023 | | 0.066 | 0.093 | 0.477 |
| **Health Insurance Status** | No *(reference)* | 37317 | | | |
| | Yes | 10127 | -0.059 | 0.016 | <0.001* |
| | Yes:2021 | | -0.001 | 0.035 | 0.981 |
| | Yes:2022 | | -0.004 | 0.027 | 0.869 |
| | Yes:2023 | | 0.000 | 0.038 | 0.998 |
| **U.S. Region of Residence** | Midwest *(reference)* | 9219 | | | |
| | Northeast | 7690 | -0.044 | 0.022 | 0.027* |
| | South | 14114 | -0.26 | 0.017 | 0.131 |
| | West | 16421 | -0.066 | 0.016 | <0.001* |

| | | | | | |
|---|---|---|---|---|---|
| | Northeast:2021 | | 0.042 | 0.051 | 0.411 |
| | Northeast:2022 | | 0.016 | 0.038 | 0.672 |
| | Northeast:2023 | | 0.080 | 0.056 | 0.153 |
| | South:2021 | | 0.051 | 0.043 | 0.228 |
| | South:2022 | | 0.014 | 0.034 | 0.684 |
| | South:2023 | | 0.095 | 0.050 | 0.056 |
| | West:2021 | | 0.085 | 0.041 | 0.040* |
| | West:2022 | | 0.005 | 0.033 | 0.889 |
| | West:2023 | | 0.080 | 0.050 | 0.106 |
| **Primary Activities** | Companion animal or pet *(reference)* | 45036 | | | |
| | Assistance or therapy dog | 335 | -0.239 | 0.072 | 0.001* |
| | Obedience | 354 | -0.044 | 0.073 | 0.546 |
| | Service Dog | 462 | -0.285 | 0.066 | <0.001* |
| | Other | 1257 | -0.007 | 0.039 | 0.865 |
| | Assistance or therapy dog:2021 | | 0.216 | 0.168 | 0.199 |
| | Assistance or therapy dog:2022 | | 0.035 | 0.135 | 0.793 |
| | Assistance or therapy dog:2023 | | 0.278 | 0.184 | 0.131 |
| | Obedience:2021 | | 0.137 | 0.165 | 0.409 |
| | Obedience:2022 | | 0.113 | 0.127 | 0.373 |
| | Obedience:2023 | | -0.067 | 0.172 | 0.698 |
| | Service Dog:2021 | | 0.146 | 0.084 | 0.083 |
| | Service Dog:2022 | | 0.026 | 0.068 | 0.697 |
| | Service Dog:2023 | | 0.085 | 0.105 | 0.418 |
| | Other:2021 | | -0.096 | 0.139 | 0.491 |
| | Other:2022 | | -0.032 | 0.109 | 0.769 |
| | Other:2023 | | -0.067 | 0.158 | 0.674 |

**4. TRAINABILITY**

| Variable | | N | Estimate | Standard Error | P-value |
|---|---|---|---|---|---|
| **Year** | 2020 *(reference)* | 27437 | | | |
| | 2021 | 5653 | -0.156 | 0.076 | 0.041* |
| | 2022 | 10379 | -0.148 | 0.063 | 0.019* |
| | 2023 | 3975 | -0.256 | 0.087 | 0.003* |
| **Breed** | Mixed *(reference)* | 23587 | | | |

| | | | | | |
|---|---|---|---|---|---|
| | Single | 23875 | -0.028 | 0.012 | 0.020* |
| | Single:2021 | | 0.045 | 0.030 | 0.128 |
| | Single:2022 | | 0.045 | 0.023 | 0.051 |
| | Single:2023 | | 0.003 | 0.034 | 0.895 |
| **Life Stage at Health and Life Experience Survey (HLES)** | Puppy *(reference)* | 2525 | | | |
| | Young Adult | 10091 | 0.365 | 0.043 | <0.001* |
| | Mature Adult | 26272 | 0.365 | 0.042 | <0.001* |
| | Senior | 8556 | -0.124 | 0.044 | 0.005* |
| | Young Adult:2021 | | 0.078 | 0.069 | 0.258 |
| | Young Adult:2022 | | 0.096 | 0.057 | 0.093 |
| | Young Adult:2023 | | 0.128 | 0.078 | 0.100 |
| | Mature Adult:2021 | | 0.038 | 0.066 | 0.566 |
| | Mature Adult:2022 | | 0.078 | 0.057 | 0.171 |
| | Mature Adult:2023 | | 0.092 | 0.077 | 0.231 |
| | Senior:2021 | | 0.136 | 0.073 | 0.062 |
| | Senior:2022 | | 0.013 | 0.062 | 0.831 |
| | Senior:2023 | | 0.067 | 0.085 | 0.429 |
| **Sex** | Male *(reference)* | 23890 | | | |
| | Female | 23554 | 0.048 | 0.012 | <0.001* |
| | Female:2021 | | -0.029 | 0.028 | 0.305 |
| | Female:2022 | | -0.031 | 0.022 | 0.172 |
| | Female:2023 | | -0.035 | 0.033 | 0.294 |
| **Spay/Neuter Status** | Intact *(reference)* | 5556 | | | |
| | Spayed/Neutered | 41888 | -0.043 | 0.027 | 0.067 |
| | Spayed/Neutered:2021 | | -0.025 | 0.047 | 0.597 |
| | Spayed/Neutered:2022 | | -0.003 | 0.038 | 0.930 |
| | Spayed/Neutered:2023 | | 0.061 | 0.055 | 0.273 |
| **Size (weight class)** | < 20lbs (~9kg) *(reference)* | 9464 | | | |
| | 21-40lbs (~10-18kg) | 9122 | 0.281 | 0.019 | <0.001* |
| | 41-60lbs (~19-27kg) | 12062 | 0.456 | 0.017 | <0.001* |
| | 61-80lbs (~28-36kg) | 10593 | 0.483 | 0.018 | <0.001* |
| | 81-100lbs (~37-45kg) | 4097 | 0.543 | 0.024 | <0.001* |
| | >100lbs (~45kg) | 2106 | 0.380 | 0.031 | <0.001* |
| | 21-40lbs:2021 | | 0.029 | 0.046 | 0.528 |

| | | | | | |
|---|---|---|---|---|---|
| | 21-40lbs:2022 | | 0.037 | 0.035 | 0.283 |
| | 21-40lbs:2023 | | 0.009 | 0.051 | 0.853 |
| | 41-60lbs:2021 | | 0.100 | 0.043 | 0.020* |
| | 41-60lbs:2022 | | 0.030 | 0.033 | 0.372 |
| | 41-60lbs:2023 | | 0.040 | 0.047 | 0.403 |
| | 61-80lbs:2021 | | 0.107 | 0.044 | 0.016* |
| | 61-80lbs:2022 | | 0.046 | 0.034 | 0.185 |
| | 61-80lbs:2023 | | 0.056 | 0.050 | 0.261 |
| | 81-100lbs:2021 | | 0.069 | 0.056 | 0.214 |
| | 81-100lbs:2022 | | 0.011 | 0.047 | 0.806 |
| | 81-100lbs:2023 | | -0.013 | 0.067 | 0.850 |
| | >100lbs:2021 | | 0.007 | 0.067 | 0.914 |
| | >100lbs:2022 | | 0.004 | 0.026 | 0.947 |
| | >100lbs:2023 | | -0.050 | 0.091 | 0.583 |
| **Health Insurance Status** | No *(reference)* | | | | |
| | Yes | 10127 | 0.028 | 0.015 | 0.069 |
| | Yes:2021 | | -0.037 | 0.035 | 0.288 |
| | Yes:2022 | | -0.023 | 0.026 | 0.375 |
| | Yes:2023 | | -0.025 | 0.037 | 0.511 |
| **U.S. Region of Residence** | Midwest *(reference)* | 9219 | | | |
| | Northeast | 7690 | -0.060 | 0.019 | 0.002* |
| | South | 14114 | -0.051 | 0.017 | 0.002* |
| | West | 16421 | -0.002 | 0.016 | 0.909 |
| | Northeast:2021 | | 0.011 | 0.050 | 0.820 |
| | Northeast:2022 | | 0.025 | 0.037 | 0.503 |
| | Northeast:2023 | | 0.037 | 0.055 | 0.629 |
| | South:2021 | | -0.007 | 0.042 | 0.975 |
| | South:2022 | | -0.015 | 0.049 | 0.648 |
| | South:2023 | | 0.061 | 0.049 | 0.211 |
| | West:2021 | | -0.021 | 0.040 | 0.597 |
| | West:2022 | | -0.009 | 0.032 | 0.786 |
| | West:2023 | | 0.012 | 0.049 | 0.809 |
| **Primary Activities** | Companion animal or pet *(reference)* | 45036 | | | |
| | Assistance or therapy dog | 335 | -0.039 | 0.071 | 0.585 |
| | Obedience | 354 | 0.278 | 0.071 | <0.001* |
| | Service Dog | 462 | -0.059 | 0.038 | 0.365 |

| | | | | | |
|---|---|---|---|---|---|
| | Other | 1257 | 0.056 | 0.038 | 0.144 |
| | Assistance or therapy dog:2021 | | 0.040 | 0.164 | 0.806 |
| | Assistance or therapy dog:2022 | | 0.154 | 0.131 | 0.242 |
| | Assistance or therapy dog:2023 | | 0.014 | 0.180 | 0.932 |
| | Obedience:2021 | | -0.100 | 0.162 | 0.555 |
| | Obedience:2022 | | -0.231 | 0.124 | 0.064 |
| | Obedience:2023 | | -0.105 | 0.168 | 0.534 |
| | Service Dog:2021 | | -0.041 | 0.136 | 0.760 |
| | Service Dog:2022 | | -0.067 | 0.107 | 0.529 |
| | Service Dog:2023 | | 0.084 | 0.155 | 0.588 |
| | Other:2021 | | 0.115 | 0.083 | 0.165 |
| | Other:2022 | | 0.101 | 0.066 | 0.126 |
| | Other:2023 | | -0.017 | 0.103 | 0.871 |
| | | | | | |
