# Supplementary material for: An analysis of behavioral characteristics and enrollment year variability in 47,444 dogs entering the Dog Aging Project from 2020 to 2023
Source: PLoS One. 2025 Sep 10;20(9):e0330257. doi: 10.1371/journal.pone.0330257 (PMC12422430; doi:10.1371/journal.pone.0330257)
Supplement: Attachment — Submitted filename: PONE Review Response June 2025_FINAL.docx [file pone.0330257.s002.docx]

PONE-D-25-10356

An analysis of behavioral characteristics and enrollment year variability in more than 47,000 dogs entering the Dog Aging Project from 2020 to 2023

PLOS ONE

**Response to Reviewers:**

**Reviewer #1:** The authors investigate changes in behavioral characteristics across 4 years of enrolled dogs in a large survey dataset. While the dataset itself is undoubtedly exciting and fruitful given its scale and scope, this manuscript ultimately falls short conceptually and methodologically. I cannot recommend it as suitable for publication at PLOS One.
My major concerns are the following:

1. Lack of clarity or compelling evidence for main claim: The central goal proposed by the authors is to create “baseline” behavioral measures for dog behavioral, but the authors do not clearly define what is meant by this baseline. Is this a baseline meant for future comparisons at the individual level? If so, I was not convinced that a separate manuscript needs to be published if there is no comparison group to each individual’s baseline presented. Or, is this meant to present group or sub-group level averages? The language used in the introduction and conclusion implies the baseline is important for tracking behavioral change, but the results presented here focus on group covariates. While I absolutely agree that understanding individuals’ baselines will be critical for future work, I was confused as to the goal of presenting them in isolation.

We appreciate this concern. At the individual level, the HLES serves as a baseline for each enrolled dog, a measure that can be compared against subsequent annual follow-up surveys submitted by participants. Our goal in this manuscript was to evaluate an overall baseline describing cohort entry year averages. This is the first group-level publication of behavioral characteristics of a long-term longitudinal study in which dogs will continue to age. With these group-level measures established, we’ll be able to track changes for the whole population over time, in addition to individual changes.

We have made an effort to clarify in the Introduction the importance of the group-level analysis (Lines 96-99; 130-134).

2. Context surrounding COVID-19 is critically underdeveloped: It seemed to me that the authors could not decide their position on how to approach potential variability introduced by data collection during the COVID 19 pandemic. The paper does not offer compelling analysis or consideration of its effects. For example, no consideration is given to whether the age at time of enrollment interacts with pandemic-related household disruptions, the age of the owner, the location of the owner, etc. A dog enrolled as a puppy in 2020 would have experienced a very different environment than a mature adult enrolled in the same year. These cohort effects deserve more thoughtful treatment, especially since the study hinges on comparisons across enrollment years.

Thank you for this comment. We have included additional context regarding the COVID-19 pandemic and its relevance to our study (Line 50; Line 77-81; Line 130; Line 205; Line 349-353).

Interaction terms were included in the model and not found to be statistically significant and so were not included in the final statistical model. Should the editors find it prudent to include these initial tests as supplement, we would be happy to provide.

3. Statistical methods are insufficiently described. The manuscript does not provide enough detail about the PCA or regression models used. No assessment of model fit and many effect sizes are left out, raising concerns about negligible effects. The authors do not describe the naming of their components or internal consistency of components. The PCA reduces 14 C-BARQ domains to four components, but the logic behind this reduction is only minimally described. Authors in the results section often report that scores are “higher” without any supporting statistical results.

Detailed descriptions of the PCA pre-analysis and PCA are included in the Results section, including domain loadings and clustering for named areas. Should the editors determine it would be more aligned with the journal standards to include this in the Methods section, we would be happy to make that adjustment.

Re: additional statistical measures, given the large number of score valuations presented in the Results section, we opted to report full results including all ns, estimates, SEs, and p-values in Table S1. It has been brought to our attention that this table was not available to reviewers in our initial submission.

4. Limited engagement with the literature and unclear contribution. The manuscript does not convincingly situate itself within existing behavioral literature. Many of the findings such as differences in behavior by size or breed are well-documented yet the authors do not identify the specific gap that their study fills. Simply stating that this is a “large” sample is not sufficient. The paper would benefit from a more focused articulation of its novelty and relevance.

We understand this concern, but the large sample size is the key component of our investigation. Our primary aim in this manuscript is to establish group baselines for a specific element of a large dataset that can be used in future analyses with the dataset. Additionally, given that a substantial amount of prior work/existing behavioral literature focuses on single breeds or draws from single institutions/sources, we did not feel it relevant to include additional background on these studies at this time.

5. No discussion or argument for CBARQ construct validity. The authors also describe behavior as a “simple” measure of emotional state, which I believe is a gross oversimplification of the nuance of animal behavior.

We have included additional information and citations regarding the validation and use of C-BARQ in various behavioral studies.

We certainly did not mean to imply that behavior is not complexed or nuanced and have removed this term.

6. Claims about relevance to human health are underdeveloped. The manuscript repeatedly refers to the importance of dogs as sentinels for human health and aging, but this idea is not explored in a meaningful way.

One of the promising directions of the Dog Aging Project is the potential to use the massive amounts of data collected about dogs who live in the same social and physical environments with their people in a translational manner in terms of investigating similar external risk factors and how they may impact health and aging in both dogs and people. We have clarified this relationship in the manuscript and included references (Lines 93-96).

Summary: While the Dog Aging Project dataset has significant potential, this manuscript does not offer a conceptually or methodologically sound analysis of that data. The framing and analytical strategy lacks clarity, and the conclusions are vague. For these reasons, I do not believe the manuscript meets PLOS ONE’s standards for publication in its current form. I encourage the authors to reconsider the framing of their research question and substantially revise their analysis and interpretive approach to understand fully the interplay of behavior and environment.

Thank you for your thoughtful insights. We have taken these and other reviewers’ suggestions into consideration and have made considerable revisions to the manuscript to clarify its purpose and findings.

**Reviewer #2:** This study is clearly described with a clear rationale, and the statistical analysis is explained in detail. My comments are limited to some very minor issues with the wording and some concerns about the discussion, which I think does not sufficiently explore the possible limitations of an owner-described dataset.

Thank you for taking the time to provide your expert review of our manuscript; we appreciate your feedback and have addressed the issues raised.

Minor issues with the wording:
line 139 - 'representative' - should this be 'represented'? - I didn't understand this.

Thank you for catching this error; it has been corrected.

line 155 - I found the frequent switching between present and past tense in this section of the methods very jolting to read. Can you reword to change tense less frequently?

We have made this adjustment.

line 387 - use of the word 'demure' - I know 'demure' was having a moment on TikTok at the time this paper was probably being written, but the definition of the word is 'reserved, modest and shy' - I don't think a dog can be modest and I think the use of this word is anthropomorphic - suggest you use 'calmer' or similar instead.

We have made this adjustment.

Concerns about the discussion - I am not familiar with the questionnaire used, so it would have been helpful to include one or two sample questions or a brief description of the sort of information that the owners were expected to provide. This study relies on owner-reported observations of canine behaviour, but we know from other research that owners are often terrible at correctly interpreting their dog's behaviour and misunderstand canine body language. You say that the shortened version of the questionnaire was validated by researchers, but then you seem to assume that the owners' reported behavioural observations are reliable without either explaining why you think this or what uncertainty this might introduce if they are not interpreting canine body language successfully. Given that many owners e.g. confuse fear and aggression and misinterpret tail movements, ear position, etc, this seems to me like a possible major weakness in the study.

We recognize that owner-reported data can be more or less reliable depending on the context. We have addressed this concern in the introduction and included additional information and citations regarding the validation and use of C-BARQ in various behavioral studies (Lines 106-111). Notably, the guide dog organization Canine Companions uses C-BARQ for generating reports from at-home puppy raisers of future service dogs.

Similarly, the discussion assumes that the judgments made by the owners are all impartial and comparable. But different types of people may choose different types of dog. Small dogs might have lower Trainability scores because they are less trainable, or perhaps their owners perceive them as less trainable or put less effort into training them than is the case for larger dog owners. Similarly, owners who do obedience with their dogs might actually have dogs with higher trainability, or might rate trainability more highly because they care enough about it to get involved with obedience! I think there needs to be at least some acknowledgement that some of this data is potentially subjective because you cannot control for the different biases that owners of different categories of dog might bring to their assessments.

We recognize that owner-reported data may introduce certain biases and have been sure to make clear that we are analyzing and interpreting owner-reported data throughout the manuscript. Given that in this report we are primarily investigating cohort year trends (as opposed to individual differences) across behavioral domains using a tool validated specifically for use in owner/non-experimenter-reported data (CBARQ) with a very large sample size (n=47,444), we do not believe these potential biases substantially impact this particular analysis, though are nevertheless interesting points for discussion and we have expanded on them in that section as suggested.

I suggest that the authors might like to look at the discussion sections of some of the owner-reported canine behavioural studies conducted by Rowena Packer (not me!) and various colleagues, because I think these do a good job of unpicking the biases that may come from owner-reported data.

Thank you for suggesting these references – very interesting work in an important arena!

In summary, I would like to see a discussion that is expanded to consider and address possible sources of bias in the data in more detail.

The discussion of these biases and potential interpretations related to them is present in our Discussion, and we have expanded the on potential relevance/import in the section (Lines 381-386; 411-415), per your recommendation.

**Reviewer #3:** The article is easy to read, but hard to interpret. I am not sure what the implications of this study and its findings are, or what problem was aimed to solve/knowledge gap was to be filled. I think the main message of the study is (/should be) that you can use the C-BARQ to identify 4 main personality domains and then which factors influence each of those domains. And DAP data was used for this. However, the focus on the article seems to be on aging and difference between years and the DAP, while that seems to be of minimal importance to what was actually done. Moreover, I don’t think the authors looked at interactions between influencing factors, while I expect there to be some. I am not an expert in the topic of statistic though, so I do not dare say whether the analysis has been performed appropriately and rigorously other than that. Nonetheless, I think the results are interesting, but I would frame the article differently to make the importance and the ‘coolness’ of your results more evident. I have provided feedback in more detail below.

Thank you for taking the time to provide your expert review of our manuscript; we appreciate your feedback and have addressed the issues raised.

Our defined aims for this study are 1) to describe behavioral data for specific characteristics for cohorts of DAP dogs at their time of enrollment; and 2) to examine behavioral data among cohorts of dogs enrolled each year over the lifetime of the project for similarities and differences and investigate additional variables that may impact observed differences. (Lines 126-131)

Abstract: It is not clear what the main rationale behind the study was, nor what your results are saying/meaning. The first part is fine, but then I got confused at the sentence ‘when considering dogs as sentinels of human health and aging’ (42-43). What do you mean with ‘sentinels’? Indicators? Or do you mean more as using dogs as models for human health? I am not a native speaker, but even a google search gave me no logic translation (‘ a soldier standing guard’). Maybe change that word to make it clearer. The second part of the sentence indicates that you probably mean it in the way that dogs can be models for humans, but then nothing else in the abstract points in the direction of this study is about using dogs as models for humans.. What does understanding dog behaviour being important for interactions and their health etc have to do with dogs being used as models for humans? And, if you do this study to say something about humans, why do the humans not come back in the last part of the abstract? Seems to me like you are focusing this project on understanding the baseline characteristics of the dogs that subscribed to the DAP, which you could potentially use later for comparison with humans/as models for humans, but that was not the main rationale here. If it was, it should be clearer. It is also unclear to me what your results implicate from reading your abstract alone. You write that time has the highest influence on trainability. In what way? Older/younger dogs becoming more/less trainable? And what does it mean that several variables are associated with mean behavioural scores? All those factors influence all behaviours? In what way? The abstract should be a summary of your article, but after reading your abstract I still know barely anything, so I would really rewrite that.

We have edited our abstract to clarify our definition of dogs as sentinels, the aims of the study, and the summary of our findings.

81-82 Here you again mention the humans (more as a side mention here in the intro then in the abstract), but it is still unclear to me why you could use dogs as models to humans. Maybe you can include some literature on how they are alike?

We have clarified this relationship and included references (Lines 93-96).

84-89 this feels like an unfinished, stand-alone paragraph, which really stops the reading flow. Maybe add some studies that used this tool and what was previously found using this tool, to link it better to the next paragraph and improve the flow in the writing. Maybe you can also link it to be previous paragraph with using a sentence like ‘A dog’s baseline behavior can be assessed with a questionnaire such as the Canine Behavioral Assessment and Research Questionnaire (C-BARQ), which is….’. However, that would mostly work if the part on understanding humans wouldn’t be in between.

We have made this adjustment.

93 It would be nice to get some background on the HLES, even if it is an added sentence like ‘, which dog owners fill in once they subscribe their dog for the DAP’.

We have made this adjustment and this information is also included in the Methods section.

101-106 On your second aim, the difference between years, there is now not really anything in the introduction, so I wonder what the relevance of this aim is? What are you trying to answer/solve her? Also, there is no aiming on using dogs as models for humans etc., so if possible I would just leave it out of your intro and abstract. If you need to put it in because of funding or similar, elaborate more on it and also put it in your aims.

One of the promising directions of the Dog Aging Project is the potential to use the massive amounts of data collected about dogs who live in the same social and physical environments with their people in a translational manner in terms of investigating similar external risk factors and how they may impact health and aging in both dogs and people. (<https://www.nature.com/articles/s41586-021-04282-9>). The aims specific to this study are part of that larger goal. Throughout the introduction we discuss behavior changes over time as being important potential health indicators, and thus the relevance to our second aim.

136 When you say the C-BARQ produces domains, you mean each dog gets a score for each of the 14 domains, or how do I see this?

The C-BARQ as a survey instrument is divided into 14 sections of questioning that correspond with distinct arenas or “domains” of behavior, as listed in Table 1.

155-160 Did you also look for interactions? I can imagine that if a certain area shows elevated scores, but a certain breed/size as well that there might be an interaction (many dogs of that kind in that area)

Interaction terms were included in the model building process and not found to be statistically significant, thus were not included in the final model. Should the editors find it prudent to include these initial tests as supplement, we would be happy to provide.

163 Was the mortality data previously described? Where?

We have removed this reference.

173-174 what test did you use to compare the means between years?

We used ANOVA to compare average behavioral score means between years and have included this in the text.

186 delete the , between dogs and the corresponding percentage

We have made this adjustment.

201-205 where these differences significant? Can you add a test statistic and p-value?

We have added p-values to Table 3.

242-248 Can you include test statistics and p-values?

These values are available in Table S1 and we have indicated this in the text.

254 I would start more general (as with fear), not directly with 2022.

We have re-structured this sentence.

259-261 It would be nice to have an reiteration of what an high attention score means. Showing more attention seeking behaviours?

These are described with our Principal Component loadings (Lines 253-261).

274-283 Add test statistics and p-values

We have added these values.

287-291 You put this as your main result in your abstract, but also here I am unsure what this means/implies. Seems to me like a coincidence since being enrolled isn’t really influencing anything for the dog? Or does this represent age of the dog..?

Our hypothesis was that due to enrollment years occurring during the Covid-19 lockdown and immediate aftermath, there may be differences in reported behaviors potentially indicative of differences in routines and attention/reports from owners.

293-300 Add test statistics and p-values

These values are available in Table S1 and we have indicated this in the text.

311-315 If your aim was to see if there were behaviour change trends in dogs, due to the Covid pandemic this should have been more evident in your introduction (talk about behaviour changes and disruptions and how earlier studies have found effects of sudden differences in contexts on the behaviour of animals, and specifically on the behaviour of dogs (eg more separation anxiety etc) and build a case from there) and I would put it as an aim. It is a cool angle and one I would really use more.

Thank you for this suggestion. We have incorporated more information to this effect in the introduction and aims (Lines 75-82; 130-134).

312 change the second ‘during’ to ‘in’

We have made this change.

331-333 Here is the first time I really understand what you mean with this result and this should really be clearer before (and makes more since in the light of the aim to find out differences due to covid years)

We have incorporated more information to clarify in the introduction and especially our Aims.

Generally discussion: I always tell my students to compare your own results with literature and then really include also what they did in that other study so it is also clear for the reader how these compare. You have a very minimal amount of other studies mentioned in your discussion and I think the story would be stronger if you would do so. There must be other studies looking into what factors influence fear, aggression, attention seeking etc., there must be other studies looking into sex or size differences etc. Did they find the same thing as you? Why (not)?

Our primary aim in this manuscript is to establish a baseline for a specific element of a large dataset that can be used in future analyses with the dataset. Given that a substantial amount of prior work/existing behavioral literature focuses on single breeds or draws from single institutions/sources, we did not feel it relevant to include additional background on these studies at this time.

Conclusion: the results are unclear again and seem to be copied from the abstract (or the other way around). The last part is strong and the abstract would also improve from an addition like that (but not identical!)

We have edited the Conclusion for clarity (and also included additional information in the Abstract).
