# Supplementary material for: An analysis of behavioral characteristics and enrollment year variability in 47,444 dogs entering the Dog Aging Project from 2020 to 2023
Source: PLoS One. 2025 Sep 10;20(9):e0330257. doi: 10.1371/journal.pone.0330257 (PMC12422430; doi:10.1371/journal.pone.0330257)
Supplement: Attachment — Submitted filename: Reviewer Report.docx [file pone.0330257.s003.docx]

Thank you to the authors for this well written study. I was invited as a reviewer to assess the revision and response to reviewers. The manuscript in well organized, well written and highlights an interesting study using the information from the Dog Aging Project.

Overall, I believe there is merit to this study being published, however there are edits I suggest to improve the manuscript. I do highly disagree with the idea of connecting this study to human health – the argument is not well constructed and lacks supporting ideas. Additionally, the study presented in this manuscript is only on dog health, which cannot be comparable. I recommend removing all mention of connections to human health and instead focus on how the results from this study can benefit dog health.

Specific Comments

Title and abstract – It would be more appropriate to list exactly how many dogs were used in the study. ‘47,444 dogs’ is different from ‘more than 40,000 dogs’ and highlights the breath of the study and the number of enrolled dogs (which is a great amount!).

Abstract – From my understanding, the journals maximum word count for the abstract is 300 words, and the abstract is currently at 308. The authors should trim down to match the journals guidelines.

L53 – I recommend removing mention of ‘human aging pattens’ since this was not analysed in this study (thus, it cannot be an aim of the study).

L54 and L83, and further instances throughout the manuscript – please use ‘COVID-19 lockdown’ vs ‘Covid lockdown’ (technically COVID itself it stands for ‘coronavirus disease 19’, and the authors should expand the acronym on first use in both the abstract and body of the manuscript).

L101 – It would be worthwhile to mention that the questions for the C-BARQ is measured on a 5-point scale.

L103 – It might be clearer for the reader if you direct them to Table 1 from here (since this is first mention of the 14 domains) but then remind them at L103 again.

Statistics – In regard to the authors response to reviewer 1, I’m of the opinion that the authors should include the initial tests (with interaction terms), in the methods (and subsequent results section). This is pertinent information that the reader should be made aware of.

L203, L226, and further instances – The journal uses SI units, and the authors should use kilograms here (in parathesis if it’s the case that DAP used lbs).

L215 – Please include citations for the packages since they are intellectual property.

Results – When the authors present percentages, directly after, they should include the numerator/denominator in parentheses.

Table 2 (and Table 3, Table 4) – I just want to mention that I really like how the authors set up this table – it’s very well organized and clear. I just recommend that the authors expand all abbreviations and define ‘n/year’. The captions should be understood without reference to the text.

I’m not convinced that this study sheds light on the human health and aging. Especially since a major result in the study relates to ‘trainability’ (which would be inappropriate to connect to humans in this context). I would advise to the authors to remove all sentences that allude to the human aging process, since none of the results in this study could be related to human aging (since I would argue human aging is much more complex with many more factors than what was presented in this study). Additionally, the authors did not explore any external factors that could impact both humans and dogs alike, rendering the conclusion that the results could translate to insight into human aging incorrect. For the authors to make this conclusion, a study would investigate both humans and dogs alike simultaneously (of which this manuscript does not accomplish).
