# Supplementary material for: An analysis of behavioral characteristics and enrollment year variability in 47,444 dogs entering the Dog Aging Project from 2020 to 2023
Source: PLoS One. 2025 Sep 10;20(9):e0330257. doi: 10.1371/journal.pone.0330257 (PMC12422430; doi:10.1371/journal.pone.0330257)
Supplement: Attachment — Submitted filename: Reviewer Response 2.docx [file pone.0330257.s004.docx]

PONE-D-25-10356R1

An analysis of behavioral characteristics and enrollment year variability in 47,444 dogs entering the Dog Aging Project from 2020 to 2023

PLOS ONE

**Response to Reviewers:**

**Reviewer #2:** The responses to the comments left by all three reviewers have considerably improved the paper's clarity and better articulated how it contributes to the literature. Looking at the other reviewers' comments, I think that the expertise of reviewer 1 in particular in statistics is much greater than mine, so I don't feel qualified to critique the statistical analysis in detail. My only remaining comment (and I apologise for not mentioning this before) is that the dogs' weights are measured and grouped in pounds. As far as I know, the USA is the only country that still uses imperial measurements in scientific publications. I'm British and familiar with both systems as I grew up with imperial, but if this paper is intended to be readily accessible to a global audience then I suggest adding kilogram equivalents to the weight bands you describe, so that international readers can appreciate these categories without having to deal with a system they have never used.

Thank you for your additional time and consideration; we are glad to hear that our revisions have improved the manuscript in your view. Thanks also for this recommendation regarding measurement units. Because the original survey was available to dog owners living in the U.S., U.S. units were provided, and we thus reported them as such for consistency. We agree, however, that it is very silly that this system is still used and have updated the manuscript to include conversions. Thank you!

**Reviewer #4:** The structure of your study is very well-designed, and the research topic itself is highly valuable. However, when transforming this study into a publishable manuscript, it is important to note that online surveys often lack reliability, particularly in studies involving animals. For instance, when classifying the dogs, the information provided by the owners or breeders may be inaccurate or unverified. Especially in the 'purebred' category, it is unclear whether the dogs have official pedigrees or not. Additionally, certain physical traits—such as categorization by size (small vs. large breeds)—cannot be confirmed through visual inspection in an online setting. Considering that the manuscript is being submitted to a Q1-ranked journal, while the study topic is indeed promising, it would be more robust and scientifically sound if the data were collected in person, with photographic documentation and visual confirmation of the animals. Such an approach would enhance the credibility and accuracy of the findings.

Thank you for your additional time and consideration. While we do recognize and note the inherent challenges of working with owner-reported data, and data validation is a core component of the Dog Aging Project. Dog Aging Project data (<https://data.dogagingproject.org/Index>) do not come from a single online survey, rather comprises a series of validated questionnaires (see McNulty et al. 2023 and Wilkins et al. 2024) completed by owners via a personalized portal, biospecimens, veterinary clinical records (which include size/weight), and additional datatypes for certain cohorts. These data have been used extensively in investigations across fields, with findings published widely and in highly ranked journals (see: <https://dogagingproject.org/publications>).

Regarding breed verification, we have a publication forthcoming (in press, *Scientific Reports*) in which we compared owner breed reporting to genetic panel results and found very high concordance for both single and mixed breed dogs.

**Reviewer #5:** Thank you to the authors for this well written study. I was invited as a reviewer to assess the revision and response to reviewers. The manuscript in well organized, well written and highlights an interesting study using the information from the Dog Aging Project.

Overall, I believe there is merit to this study being published, however there are edits I suggest to improve the manuscript. I do highly disagree with the idea of connecting this study to human health – the argument is not well constructed and lacks supporting ideas. Additionally, the study presented in this manuscript is only on dog health, which cannot be comparable. I recommend removing all mention of connections to human health and instead focus on how the results from this study can benefit dog health.

All line numbers refer to the tracked change version, which I’ve included as an attached Word Document.

Thank you very much for taking the time to provide such thoughtful feedback. We have taken your comments and the suggestions of other reviewers to heart and have reconsidered our inclusion of the sentinel narrative (connections to human health) in this manuscript. We have likewise addressed your additional comments below.

Specific Comments

Title and abstract – It would be more appropriate to list exactly how many dogs were used in the study. ‘47,444 dogs’ is different from ‘more than 40,000 dogs’ and highlights the breath of the study and the number of enrolled dogs (which is a great amount!).

We have made this change.

Abstract – From my understanding, the journals maximum word count for the abstract is 300 words, and the abstract is currently at 308. The authors should trim down to match the journals guidelines.

We have revised the abstract and ensured it is compliant with word count.

L53 – I recommend removing mention of ‘human aging pattens’ since this was not analysed in this study (thus, it cannot be an aim of the study).

We have made this change.

L54 and L83, and further instances throughout the manuscript – please use ‘COVID-19 lockdown’ vs ‘Covid lockdown’ (technically COVID itself it stands for ‘coronavirus disease 19’, and the authors should expand the acronym on first use in both the abstract and body of the manuscript).

We have amended to the proper nomenclature.

L101 – It would be worthwhile to mention that the questions for the C-BARQ is measured on a 5-point scale.

We have included mention of the scale.

L103 – It might be clearer for the reader if you direct them to Table 1 from here (since this is first mention of the 14 domains) but then remind them at L103 again.

We have included a reference to Table 1 here.

Statistics – In regard to the authors response to reviewer 1, I’m of the opinion that the authors should include the initial tests (with interaction terms), in the methods (and subsequent results section). This is pertinent information that the reader should be made aware of.

We have updated the Methods and Results sections to include the initial tests and equations and have provided the results from the interaction terms model in an additional supplementary table (Table S2).

L203, L226, and further instances – The journal uses SI units, and the authors should use kilograms here (in parathesis if it’s the case that DAP used lbs).

Yes! Another reviewer just flagged that as well. The DAP surveys are in lbs. but we have updated to include the conversions in parentheses.

L215 – Please include citations for the packages since they are intellectual property.

We have included these citations.

Results – When the authors present percentages, directly after, they should include the numerator/denominator in parentheses.

Where percentages are given, we already indicate in the text the total number that the percentages are out of, and the numerical totals are present in Table 2 so we have opted to keep these reported as percentages.

Table 2 (and Table 3, Table 4) – I just want to mention that I really like how the authors set up this table – it’s very well organized and clear. I just recommend that the authors expand all abbreviations and define ‘n/year’. The captions should be understood without reference to the text.

Thank you very much! And we have made these adjustments.

I’m not convinced that this study sheds light on the human health and aging. Especially since a major result in the study relates to ‘trainability’ (which would be inappropriate to connect to humans in this context). I would advise to the authors to remove all sentences that allude to the human aging process, since none of the results in this study could be related to human aging (since I would argue human aging is much more complex with many more factors than what was presented in this study). Additionally, the authors did not explore any external factors that could impact both humans and dogs alike, rendering the conclusion that the results could translate to insight into human aging incorrect. For the authors to make this conclusion, a study would investigate both humans and dogs alike simultaneously (of which this manuscript does not accomplish).

We have removed this narrative thread throughout. Thank you.
